# Targeting super-enhancers in liver cancer: from pathogenic mechanisms to clinical applications

**DOI:** 10.3389/fphar.2025.1589455

**Published:** 2025-06-18

**Authors:** Chang-Lei Li, Zhi-Yuan Yao, Ao Sun, Jing-Yu Cao, Zu-Sen Wang

**Affiliations:** ^1^ Department of Hepatobiliary and Pancreatic Surgery, The Affiliated Hospital of Qingdao University, Qingdao, Shandong, China; ^2^ Department of Thoracic Surgery, The Affiliated Hospital of Qingdao University, Qingdao, Shandong, China

**Keywords:** liver cancer, super-enhancer, transcriptional regulation epigenetic modifications, therapeutic targets, clinical applications

## Abstract

Liver cancer, especially primary liver cancer (PLC), stands as the third leading cause of cancer-related mortality globally, posing a significant threat to human health. Super-enhancers (SEs), clusters of enhancer elements with high histone modifications and transcriptional activity levels, play crucial roles in diverse biological processes and are closely associated with the pathogenesis of various diseases, including liver cancer. This review first delves into the pathogenic mechanisms of super - enhancers in liver cancer. SEs can drive the aberrant expression of oncogenes in liver cancer. Through interactions with transcription factors and chromatin-modifying enzymes, SEs can reshape the chromatin architecture, facilitating the access of transcriptional machinery to oncogene promoters and resulting in their overexpression. Additionally, abnormal activation of signaling pathways in liver cancer can also regulate the formation and activity of SEs, creating a positive - feedback loop that fuels tumor development. We further explore how targeting SEs may translate into clinical applications for liver cancer. Therapeutic strategies, such as using small inhibitors that disrupt the function of key components in SE-mediated transcriptional complexes, have shown promise in pre-clinical studies. These inhibitors can specifically block the activity of SEs, leading to the downregulation of oncogene expression and subsequent suppression of tumor cell growth. In addition, gene-editing technologies provide new tools for precisely modulating super-enhancer activity in liver cancer cells. By deleting or modifying specific enhancer elements within SEs, the expression of oncogenes can be effectively controlled. In conclusion, understanding the pathogenic mechanisms of SEs in liver cancer and their clinical applications offers a new perspective on the diagnosis, treatment, and prognosis of liver cancer. However, more in-depth research is required to fully realize the potential of super-enhancer-targeted therapy in clinical settings in order to provide more effective treatment options for liver cancer patients.

## 1 Introduction

Primary liver cancer (PLC) is the third leading cause of cancer-related deaths, representing a major global health challenge (Sung et al., 2021). Accordingly, PLC mainly comprises three pathological types: hepatocellular carcinoma (HCC), intra-hepatic cholangiocarcinoma (ICC), and a specific classification—combined hepatocellular-cholangiocarcinoma (CHC) (Sia et al., 2017; Seehawer et al., 2019). Nowadays, the common causes of liver cancer primarily include alcohol-associated liver disease (ALD), metabolic dysfunction-associated steatotic liver disease (MASLD), chronic hepatitis B virus (HBV) infection, and chronic hepatitis C virus (HCV) infection (Choi et al., 2023). Liver cancer is predominantly diagnosed in middle-aged and elderly adults, with a median age of diagnosis being 62. Nevertheless, 14.7% of incident cases are observed in younger adults ranging from 15 to 49 years of age (Danpanichkul et al., 2024; Yang et al., 2017). More concerningly, in the past two decades, incidences of liver cancer elevated by 53.7%, while the death incidence increased by 48.0% (Naghavi et al., 2024). Despite improvements over time, the prognosis for liver cancer remains poor, with 5-year overall survival rates of less than 20%, mainly due to late-stage diagnosis, unpredictive recurrence, and advanced metastasis (Huang et al., 2023). Importantly, current therapeutic strategies, especially surgical resection, local ablation, neoadjuvant and adjuvant treatment, trans-arterial chemoembolization (TACE), trans-arterial radioembolization (TARE) and even liver transplantation, provide limited survival benefits, notably in advanced the liver cancer (Hwang et al., 2024).

The pathogenesis of liver cancer is a complex multistep process that involves sustained inflammatory damage, mainly exhibited by hepatocyte necrosis and regeneration, concurrently associated with fibrotic deposition. The underlying mechanism is also intricate, involving genetic mutations, epigenetic modifications, and changes in cellular signaling pathways that regulate cell growth, apoptosis, and metabolism (Llovet et al., 2021; Schulze et al., 2016). Transcriptional regulation has emerged as a critical promoter of HCC progression among these regulatory mechanisms (Niu et al., 2021). The ability of malignant cells to harass transcriptional landscapes that promote uncontrolled proliferation and invasion reveals the ability of the malignancy’s aggressiveness.

Enhancers were first identified in the Simian virus 40 genome and are a cluster of short DNA sequences that can elevate the transcription efficiency of target genes (Banerji et al., 1981). Unlike promoters regulating a proximal partner, typical enhancers (TEs) can activate the transcription of their target genes irrespective of genomic orientation and distance limitation (Bulger and Groudine, 2011). Intriguingly, the human genome also contains a large cluster of consecutive TEs, which were defined as super-enhancers (SEs), with the nature of close genome proximity (regions cover 5–12.5 kb) (Hnisz et al., 2013; Whyte et al., 2013). SEs can bind the mediator complex (MED), various transcription factors (TFs), and even the RNA polymerase II complex (pol II) at significantly high densities (Hnisz et al., 2015). Recent research has reported that SEs play a vital role in the pathogenesis of several cancer types, notably in pancreatic cancer, lung cancer, gastric cancer, colorectal cancer (CRC), and breast cancer (Lovén et al., 2013; Akhtar-Zaidi et al., 2012; Pasquali et al., 2014; Parker et al., 2013; Wei et al., 2024; Yao et al., 2024; Guo et al., 2024; He et al., 2024). Pathogenic genomic changes, such as chromosomal amplifications, chromosomal translocations, and chromosomal insertions/deletions (indels), disturb SEs and alter gene expression (Christensen et al., 2014; Chapuy et al., 2013; Mansour et al., 2014).

Nowadays, the pivotal roles of SEs in the pathogenesis and progression of liver cancer have become a hot topic (Chen J. et al., 2023; Liu Z. et al., 2022; Peng et al., 2022). Regarding their critical role in modulating the expression of oncogenes and their effects on tumor progression, SEs have been perceived as key players in the pathogenesis of liver cancer. Also, identifying SEs in liver cancer has provided novel opportunities for targeted therapies. Thus, this review aims to explore the role of SEs in liver cancer, particularly in HCC. We will examine the mechanisms by which SEs contribute to liver cancer etiology, highlighting their impact on critical oncogenes and associated cellular pathways. Furthermore, we will discuss the potential of targeting SEs as a therapeutic strategy and the challenges and limitations researchers must face to make SEs-based personalized therapies a clinical reality.

## 2 The biology of super-enhancers

SEs are large and multi-kilobase regions of the genome that cover multiple enhancers. SEs are commonly located in the SE domains, specifically within the topologically associating domain (TAD), an organized eukaryotic genome structure affected by the chromatin three-dimensional (3D) conformation (Wang et al., 2023; McArthur and Capra, 2021). Furthermore, SEs activate the gene expression that controls cell identity, employ a large assortment of transcriptional elements to elevate the overexpression of the corresponding genes, and are primarily enriched in disease-related mutations (Blobel et al., 2021). SEs are distinct from regular enhancers due to their size, complexity, and functional importance. While typical enhancers regulate the expression of one or a few genes, SEs can influence the expression of key genes essential for cell-type-specific functions, particularly in sustaining cell identity.

### 2.1 Definition and signatures of super-enhancers

Enhancer was first identified on a 72 bp sequence by Banerji et al., in 1981 in the SV40/hemoglobin β1 recombinant gene genome, accompanied by the elevated expression of the rabbit β globin gene (Banerji et al., 1981). Recently, SEs were first described by Hnisz et al., in 2013 Hnisz et al. (2013), compared to regular enhancers, enriching more in cell-specific genes and featuring a higher density of TFs, coactivators, and chromatin modifiers. A significant feature of SEs is their large size—they surround kilobases of DNA, distinct from smaller enhancers. Besides, SEs interact with a denser accumulation of master TFs (e.g., Oct4, Nanog, Sox2) and are associated with co-factors such as Mediator Subunit 1 (Med1) and p300 (Hnisz et al., 2017). SEs are marked with active histone modifications—H3K4me1 (histone H3 lysine four mono methylation) and H3K27ac (acetylation of histone H3 at lysine 27), chromatin modulators—BRD4 (Bromodomain-containing protein 4), CDK7 (cyclin-dependent kinase 7) and RNA Pol II (Lakhia et al., 2023; Barral and Déjardin, 2023; Yoo et al., 2019). Furthermore, SEs possess an enhanced capacity to drive transcription and increased vulnerability to perturbation. For example, Whyte et al. notably observed a significant reduction in SE-associated gene expression following the loss of pluripotency in mouse embryonic stem cells, which occurred after the knockdown of OCT4, the key TF responsible for regulating this specific SE (Whyte et al., 2013). After Chen et al. pointed out the concept of a SE (Chen et al., 2004), Young et al. created a new method to identify SEs; while at this time, most studies on transcriptional regulatory elements of genes were limited to transcription start sites (TSSs) (Lee and Young, 2013).

However, we have summarized that SEs exhibited several key signatures: biomarker composition, structural uniqueness, functional hierarchy, dynamic regulation, and even cancer association. Herein, we will probe into these perspectives in the following parts, especially the role of SEs in liver cancer.

### 2.2 Two types of SEs’ working models

#### 2.2.1 Typical model of SE activation

**FIGURE 1:**
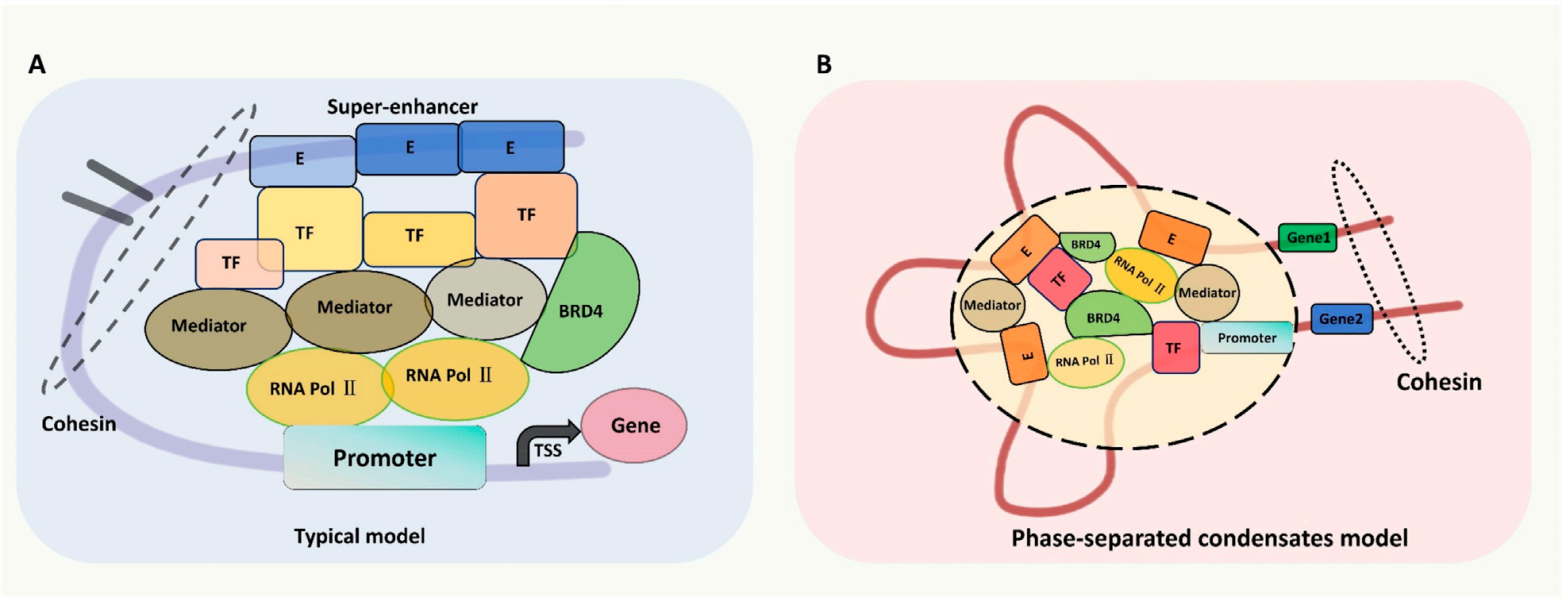
Two types of SEs’ working models: **(A)** typical model and **(B)** phase-separated condensates model. **(A)** Typical Model of SE Activation: This model illustrates the sequential recruitment of pioneer transcription factors (TFs) to open chromatin, followed by coactivators (e.g., histone acetyltransferases, MED complex). The MED complex facilitates chromatin looping, enabling SE-promoter interactions for transcriptional activation of oncogenes (e.g., MYC, AJUBA). Key signatures include high-density histone modifications (H3K4me1, H3K27ac) and transcriptional machinery (BRD4, RNA Pol II). **(B)** Phase-Separated Condensate Model: Liquid-liquid phase separation (LLPS) mediated by multivalent interactions between TFs, MED1, and BRD4 enables SE assembly into transcriptional condensates. These membraneless compartments concentrate transcriptional machinery to amplify oncogene expression. In liver cancer, perturbations in LLPS (e.g., BRD4/CDK7 overexpression) enhance SE-driven transcription of proliferation and survival genes. Targeting phase separation components (e.g., BRD4 inhibitors) disrupts condensate stability, suppressing tumor growth.

Firstly, the model engages pioneering TFs—unique DNA-binding proteins that access nucleosomal DNA to open chromatin—then recruits coactivators including histone modifiers (e.g., acetyltransferases), ATP-dependent remodelers, or MED (Calo and Wysocka, 2013; Larson et al., 2021). DNase I sensitivity assays can assess DNA accessibility, which is mediated by TFs and chromatin remodelers, especially for those active-transcribed related genes (Ramachandran and Henikoff, 2016). Next, the high density of MED, in conjunction with the pre-initiation complex, facilitates long-distance chromatin interactions and the formation of 3D loops. In this 3D chromatin architecture, the complex activates transcription by interacting with RNA polymerase II and binding to the promoters of target genes. The model is further refined by suggesting that MEDs tightly associate with cell type-specific regulatory regions of genes, including enhancers and promoters (Levine et al., 2014; Kagey et al., 2010; Figure 1A).

#### 2.2.2 Phase-separated condensate model

The other pivotal model is associated with liquid-liquid phase separation (LLPS), contributing to SE assembly and transcriptional regulation (Hnisz et al., 2017; Sabari et al., 2018; Tang, 2019; Hyman et al., 2014). Mechanistically, SEs harbor a dense cluster of polymers, necessitating a more stable structural framework to preserve their functionality and prevent disintegration. LLPS has emerged as the predominant mode of interaction between membrane-less organelles, addressing this requirement. Sabari et al. demonstrated that MED1, a core component of the MED complex, and BRD4, a member of the BET protein family, are both essential for phase-separated condensate formation. MED1 operates as an integral subunit of the MED complex, while BRD4 interacts with acetylated chromatin *via* its bromodomains. These two factors synergistically regulate the transcriptional activity of super-enhancers (Hyman et al., 2014). This model’s enhancer constituents and multivalent proteins, including TFs, MED, and BRD4, enhance phase-separated multimolecular assemblies and compartmentalize transcriptional processes (Hnisz et al., 2017). LLPS-derived condensates promote the exchange and interaction of various components, thereby supporting the functional activity of SEs (Brangwynne et al., 2009; Figure 1B).

## 3 Super-enhancers in liver cancer

**FIGURE 2:**
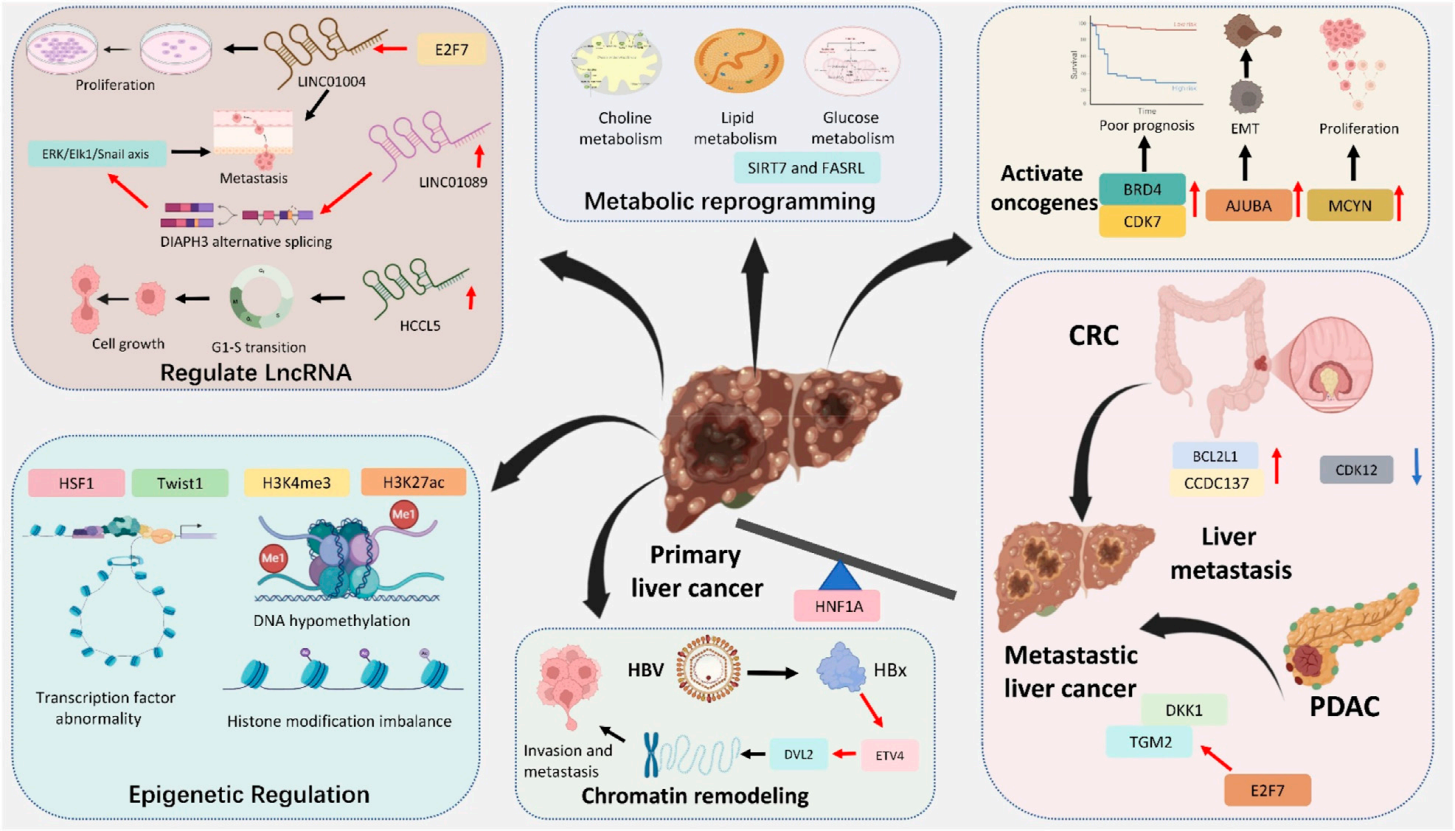
Major roles of Super-Enhancers in liver cancer: activate oncogenes (BRD4, CDK7, AJUBA and MCYN), regulated LncRNA (LINC1004, LINC1089 and HCCL5), metabolic reprogramming (choline, lipid and glucose metabolism), epigenetic regulation (transcription factor abnormality, DNA hypomethylation and histone modification imbalance), chromatin remodeling and promote liver metastasis (from CRC and PDAC).

Liver cancer, especially HCC, is a complex malignancy driven by genetic, epigenetic, and transcriptional alterations (Schulze et al., 2016; Llovet et al., 2021). Accordingly, increasing evidence indicates that SEs regulate cancer characteristics, mainly including epithelial-mesenchymal transition (EMT), cell proliferation, metabolic reprogramming, and even cancer metastasis (Zhang et al., 2020; Ng et al., 2020). Recent studies have identified SEs as crucial players in the molecular landscape of liver cancer. SEs contribute to the transcriptional control of ncRNAs, such as primary microRNAs (pri-miRNAs), not limited to influencing the protein-coding genes. These regions regulate the expression of oncogenes and genes involved in essential cancer hallmarks, such as uncontrolled proliferation, metabolic reprogramming, immune evasion, and metastasis (Cai et al., 2021; Liang et al., 2021; Wu et al., 2022; Wu et al., 2024). This part summarizes the SE landscapes in liver cancer and the SE-related target genes in liver cancer (Figure 2; Table 1).

**TABLE 1:** Key components and functional roles of SEs in liver cancer.

Key components	Functional roles	Mechanisms/targets	Clinical significance	References
1. Oncogene activation				
CDK7, BRD4	Drive transcriptional amplification of oncogenes	Overexpression correlates with poor prognosis; BRD4 interacts with SEs	Potential therapeutic targets (e.g., AZD5153 disrupts BRD4-SE interaction)	Lin et al. (2022)
AJUBA	Promotes EMT and metastasis	SE-associated transcriptional coactivator	Linked to aggressive tumor behavior	Zhang et al. (2020)
MYC family	Regulates tumor cell proliferation and metabolic reprogramming	Binds SEs to activate genes in cell cycle and glycolysis	Poor prognosis marker; potential druggable axis	Liu et al. (2023)
2. Metabolic Reprogramming				
SIRT7, FASRL	Modulates lipid metabolism and mitochondrial dysfunction	SEs regulate genes in glucose/lipid metabolism and oxidative stress	Links hyperlipidemia to HCC progression; therapeutic vulnerability	Wu et al. (2022), Gu et al. (2024), Shin et al. (2013), Yoshizawa et al. (2014)
Choline metabolism genes	Altered choline pathways in tumor microenvironment	SE-driven transcriptional dysregulation	Potential biomarker for early detection	Li et al. (2024b)
3. Immune Evasion				
PD-1/PD-L1 axis	Facilitates immune checkpoint activation	SE-mediated transcriptional control of immune-related genes; prognostic model based on SE-related genes, higher infiltration level of PD1-positive immune cells	Biomarker for response to checkpoint inhibitors; Prediction for prognosis	Wei et al. (2023)
4. Promote metastasis				
DHX37	Promote continuous cell division and tumor growth	Knockdown of DHX37 restricted the HCC cell proliferation, which was deemed as one of the top RNA helicase-related upregulated genes in HCC.	A potential therapeutic target in HCC	Liu et al. (2022a)
SE-related LncRNAs	SE-lncRNAs can interact with their associated enhancer regions in cis and modulate oncogenes or key signal pathways	HCCL5 as a crucial oncogene in HCC, promoting G1-S transition, cell growth, invasion, and even metastasis; LINC01004 promoted cell proliferation and metastasis of HCC, mediated by of E2F1 to the SE; LINC01089 forms an epigenetic network to promote the HCC metastasis	Potential therapeutic target in primary liver cancer and HCC metastasis	Peng et al. (2019a)
5. Epigenetic Regulation				
H3K4me3, H3K27me3	Marks active SE regions; induce DNA methylation and histone modification imbalances	H3K4me3 promotes open chromatin and RNA Pol II recruitment at SE-driven oncogenes, while H3K27me3 may fine-tune activation thresholds to prevent genomic instability	Therapeutic target for BET inhibitors	Chen et al. (2023a), Liu et al. (2024b), Wang et al. (2024)
HSF1, Twist1	Transcriptional hijacking of SE networks	Abnormal TF binding reshapes SE-driven transcriptional programs	Prognostic indicators for metastasis	Liu et al. (2024b), Meng et al. (2023)
6. Chromatin Remodeling				
ETV4, HBx	Mediates chromatin looping in HBV-associated HCC	HBx upregulates ETV4 to facilitate SE-mediated chromatin remodeling|	Therapeutic focus for virus-driven HCC	Zheng et al. (2022)

### 3.1 Overview of liver cancer molecular landscape

Liver cancer develops in the landscapes of chronic liver injury, commonly ascribed to viral hepatitis (HBV/HCV), alcoholic liver disease, or NAFLD. Concerningly, the progression from cirrhosis to liver cancer is often slow but significantly accelerates based on additional genetic and epigenetic alterations (Estes et al., 2018). These alterations drive the dysregulation of several vital signaling pathways, such as Wnt/β-catenin, PI3K/Akt, MAPK/ERK, and Notch, in addition to genetic mutations in TP53, CTNNB1 (β-catenin), and AXIN1 that can disrupt normal cellular processes, notably for cell cycle regulation, apoptosis, and cell adhesion (He and Tang, 2020; Sun et al., 2022; Liu W. et al., 2024; Kawaguchi and Kaneko, 2021; Calderaro et al., 2019; Xu et al., 2022).

One of the hallmarks of liver cancer is the altered transcriptional regulation of genes involved in these pathways. SEs often regulate these genes, which cluster in regions that control multiple pivotal oncogenes (Tsang et al., 2019). Notably, genes corresponding to cell proliferation, metabolism, and survival are regulated by SEs reprogrammed during tumorigenesis, driving the malignant transformation of normal hepatocytes.

### 3.2 Role of super-enhancers in liver cancer

In liver cancer, SEs are essential in upregulating oncogenes and other related genes that sustain the cancer cells’ malignant characteristics. Important points where SEs contribute to liver cancer pathogenesis include.

#### 3.2.1 Activation of oncogenes

In 2019, Wong’s group first described aberrant SE landscapes in human HCC, demonstrating that the cis-acting SE landscape was substantially reprogrammed during liver carcinogenesis (Tsang et al., 2019). They further identified several critical compositions of the trans-acting SE complex—CDK7, BRD4, EP300, and MED1-that were frequently overexpressed in human HCCs and were associated with the poor prognosis of patients with HCC (Zhang et al., 2019; Pei et al., 2023; Chang et al., 2019; Chen et al., 2021). Furthermore, concurrent co-overexpression of BRD4 and CDK7 accelerates cell proliferation and suggests poor prognosis in HCC (Li et al., 2024a). Additionally, in acute myeloid leukemia, inhibition of BRD4 disrupts MYC-driven SEs but can lead to compensatory activation of parallel SEs regulating alternative oncogenes like BCL2 or MCL1, promoting survival (Fiskus et al., 2014; Tzelepis et al., 2018). While in neuroblastoma, targeting CDK7 suppresses MYCN expression but may trigger rewiring of stress-response pathways to sustain proliferation (Durbin et al., 2018). Similarly, JMJD6-related pathway was also involved in the CDK7/SEs regulation process (Wong et al., 2019).

In 2020, Xie’s group performed integrated analysis of ChIP-seq and Hi-C data in HCC cells, identifying the ajuba LIM protein (AJUBA) as an SE-associated gene (Zhang et al., 2020). Their experimental data revealed that transcriptional factor 4 (TCF4) directly regulates AJUBA expression through binding to its super-enhancer region. Mechanistically, AJUBA overexpression was shown to interact with tumor necrosis factor receptor-associated factor 6 (TRAF6), facilitating Akt phosphorylation and subsequent induction of epithelial-mesenchymal transition (EMT) in HCC cells.

Lin et al. employed ChIP-seq to demonstrate that AZD5153 (a bifunctional BRD4 inhibitor) significantly reduces BRD4 binding at super-enhancers (SEs), promoters, and gene bodies in HCC cells (Lin et al., 2022). Subsequent RNA-seq analysis revealed that this BRD4 displacement led to transcriptional repression of key target genes involved in DNA replication, cell cycle progression, and anti-apoptotic signaling pathways.

MYC family, a cluster of significant oncogenic TFs in many malignancies, including liver cancer (Tzelepis et al., 2018), acts as a central player in multiple pathways, such as cell proliferation and metabolism. Importantly, *in vivo* experiments, the MYC pathway can dysregulate the expression of dedifferentiation genes in liver tumors. At the same time, MYC-positive HCC patients are more likely to exhibit positive expression of AFP in clinical samples (Watanabe et al., 2019). Recently, Liu et al. reported that heat shock transcription factor 1 (HSF1) stimulates liver cancer cell proliferation both *in vitro* (Huh7 cell line) and *in vivo* (BALB mice, female), owing to transcriptionally activating MYCN expression by combining with its promoter and SE elements (Liu Y. et al., 2024). In addition, after applying a genome-scale CRISPR knockout algorithm, the SET domain containing 1A (SETD1A) was identified by Chen et al., which was inversely associated with the clinical prognosis in HCC patients. In this study, SETD1A-mediated transcriptional activation of histone-modifying enzymes leads to the deposition of both H3K4me3 (an activating mark) and H3K27me3 (a repressive mark) at SEs. H3K4me3 promotes open chromatin and RNA Pol II recruitment at SE-driven oncogenes, while H3K27me3 may fine-tune activation thresholds to prevent genomic instability. Also, H3K27me3 could influence 3D chromatin architecture by stabilizing SE-promoter loops or defining topological domain boundaries (Chen J. et al., 2023). Also, the knockdown of SETD1 augmented proliferation repression and cell death caused by sorafenib (Wu et al., 2020).

Long non-coding RNAs (lncRNAs) can also be activated by SEs in HCC. Notably, Lin’s group identified a novel lncRNA, HCCL5, as a pivotal oncogene in HCC that drives G1-S phase transition, cell proliferation, invasion, and metastasis (Peng L. et al., 2019). Transcriptionally, HCCL5 is regulated by ZEB1 through binding to an SE region, resulting in its significant overexpression in HCC tissues, which correlates with enhanced malignancy. Mechanistically, HCCL5 potently induces the EMT phenotype in HCC cells by upregulating transcription factors including Snail, Slug, ZEB1, and Twist1.

#### 3.2.2 Metabolic reprogramming

The onset and progression of liver cancer are often accompanied by metabolic reprogramming, which is crucial for promoting cancer cell proliferation and creating barriers to the anticancer immune response and limited durable clinical remission following immunotherapy. Lin et al. previously reviewed the underlying metabolic communication between liver cancer cells and their surrounding immune cells, altering the landscapes of the immune microenvironment (Lin et al., 2024). SEs regulate the expression of genes involved in metabolic reprogramming, including those responsible for glucose metabolism, lipid biosynthesis, and mitochondrial impairment (Liu K. et al., 2022; Guo et al., 2023; Zhang H. et al., 2022). In HCC, elevated SIRT7 expression is correlated with increasing grade, robustly indicating that the upregulation of SIRT7 contributes to a higher malignant HCC phenotype (Gu et al., 2024). Moreover, inhibiting the expression of SIRT7 has been validated to aggregate hepatic gluconeogenesis, steatohepatitis, and lipid metabolism (Shin et al., 2013; Yoshizawa et al., 2014). Through integrating epigenomic profiling of NAFLD-associated HCCs, Wu et al. revealed a compendium of SE-activated chromatin regulators, pointing out that Sirtuin 7 (SIRT7) SE-driven tumor-suppressor silencing is associated with metabolic and immune dysfunction for tumor progression (Wu et al., 2022). Additionally, in lung cancer, disruption of SIRT7-ARF signaling stabilizes ARF and thus attenuates cancer cell proliferation, providing a strategy to mitigate NSCLC progression (Kumari et al., 2024).

In addition to modulating protein-coding genes, the role of SEs in regulating non-coding genes in HCC lipid metabolism is equally essential. Recently, Peng’s group identified a novel lncRNA, named fatty acid synthesis-related lncRNA (FASRL), through binding to acetyl-CoA carboxylase 1 (ACACA) to increase fatty acid synthesis and lipid accumulation and finally exacerbate HCC (Peng et al., 2022). ACACA is also a significant lipid metabolism enzyme and the key enzyme controlling *de novo* fatty acid (FA) biosynthesis (Hunkeler et al., 2018). By chance, the expression of FASRL is activated by its upstream stimulatory factor 1 (USF1) *via* its SE. This research also demonstrated the higher expression of FASRL, USF1, and ACACA corresponds with a worse prognosis in HCC patients.

Lipid metabolism dysregulation has been considered a risk factor for HCC, simultaneously indicating the prognosis (Du et al., 2022; Pope et al., 2019). Thus, probing into the profiling of fatty liver disease is necessary for liver cancer prevention. Hu and colleagues delved into the fundamental role of SEs in developing hyperlipidemia (HLP). Their findings suggest that HLP may be potentially triggered by a pathogenic regulatory network of hepatic SEs under a high-fat diet (HFD). Among the identified SEs, 278 were recognized as HFD-specific SEs (HSEs). Gene Ontology (GO) and Kyoto Encyclopedia of Genes and Genomes (KEGG) pathway enrichment analyses of the genes associated with upregulated HSEs revealed that these genes predominantly participated in the lipid metabolic pathway. In the protein-protein interaction network associated with HSEs, four hub genes, namely, Cd36, Pex11a, Ech1, and Cidec, were pinpointed. The authenticity of these hub genes was further confirmed using two additional datasets (Hu et al., 2024). Additionally, Zhu’s group established a high-fat diet-induced fatty liver hemorrhage syndrome (FLHS) chicken model to investigate the genome-wide active enhancers and transcriptome by H3K27ac target chromatin immunoprecipitation (Wang et al., 2024). Intriguingly, the PCK1 gene was substantially covered in upregulated SEs, which further implied the vital role of PCK1 during the development of FLHS.

Oxidative stress response (ROS) accumulation has been verified to promote the proliferation of HCC cells (Zhu Y. et al., 2024; Yang R. et al., 2024). Liu et al. analyzed the GSE112221 dataset using HOMER to identify SEs, followed by functional enrichment analysis of SE-regulated genes *via* Metascape. This approach identified 318 HCC-specific SE-associated genes showing significant functional correlation with ROS pathways. SPIDR and RHOB were enriched in the “response to oxidative stress” category and selected for experimental validation. Genetic silencing of SPIDR or NRF1 significantly enhanced ROS accumulation in HCC cells. Under oxidative stress conditions, knockdown of these genes increased intracellular ROS, malondialdehyde, and γH2AX levels, while reducing superoxide dismutase (SOD) activity and suppressing HCC cell proliferation (Liu B. et al., 2024). Emerging evidence suggests that metabolites generated through SE-driven pathways can reciprocally modulate SE activity. For example, α-ketoglutarate (α-KG), a TCA cycle intermediate upregulated by SE-activated *IDH2*, serves as a cofactor for TET enzymes and histone demethylases, thereby remodeling chromatin accessibility and potentiating SE-dependent transcription (Shin et al., 2013). Conversely, accumulation of ROS due to SE-enhanced mitochondrial dysfunction activates stress-responsive TFs (e.g., NRF2), which bind SE regions to further amplify pro-survival gene expression (Yoshizawa et al., 2014). These feedback loops create a self-reinforcing circuit that stabilizes the oncogenic metabolic state in HCC.

Aberrant choline metabolism in cancer is closely associated with malignant progression (Yao et al., 2023). Mechanistically, choline supplementation elevates S-adenosylmethionine (SAM) levels, which induces H3K4me1 deposition within the SE region of KLF5, thereby activating its transcriptional output. Functioning as a choline-regulated master transcriptional hub in HCC, KLF5 drives tumor cell cycle progression through transactivation of downstream effectors. Furthermore, KLF5 upregulates choline kinase-α (CHKA) and CTP: phosphocholine cytidylyltransferase (CCT), establishing a self-reinforcing choline metabolism-epigenetic circuitry that sustains HCC proliferation. Pharmacological inhibition using the histone deacetylase inhibitor vorinostat effectively attenuates KLF5 expression, impedes hepatic tumorigenesis in murine models, and prolongs survival duration. These results delineate a SE-mediated epigenetic mechanism whereby choline metabolism governs HCC pathogenesis *via* KLF5 activation (Li et al., 2024b). Similarly, SEs-driven KLF5 is a key regulatory factor in ovarian cancer progression and PARPi resistance; offering potential therapeutic strategies for these patients with PARPi resistance and high KLF5 are identified (Wu Y. et al., 2023).

Recent studies have revealed that SEs not only regulate individual metabolic pathways but also orchestrate crosstalk between them. For example, MYC, a SE-driven oncogene in HCC, simultaneously activates glucose transporter 1 (GLUT1) to enhance glycolysis and upregulates ATP-citrate lyase (ACLY) to promote lipid biosynthesis by converting glycolytic intermediates into acetyl-CoA (Liu Y. et al., 2024; Guo et al., 2023). This metabolic coupling ensures a steady supply of acetyl-CoA for both energy production and epigenetic modifications (e.g., histone acetylation), thereby sustaining proliferative signaling. Additionally, SE-mediated overexpression of SETD1A enriches H3K4me3 modifications at promoters of genes involved in both gluconeogenesis (PCK1) and fatty acid oxidation (CPT1A), linking glucose deprivation to lipid catabolism under metabolic stress (Chen J. et al., 2023). Such coordination highlights the role of SEs as “metabolic hubs” that integrate nutrient availability with transcriptional outputs to drive HCC progression.

Importantly, SE-driven metabolic reprogramming extends beyond cell-autonomous effects to shape the immunosuppressive tumor microenvironment. For instance, SE activation of IDO1 (indoleamine 2,3-dioxygenase 1) promotes tryptophan catabolism, leading to kynurenine accumulation that suppresses CD8^+^ T cell function (Zhang H. et al., 2022). Concurrently, SE-mediated upregulation of PD-L1 in HCC cells is fueled by enhanced aerobic glycolysis, which provides ATP and metabolites necessary for immune checkpoint protein synthesis (Gu et al., 2024). These findings illustrate how SEs synchronize metabolic adaptations with immune evasion mechanisms, further underscoring the need for therapeutic strategies that target SEs to disrupt both oncogenic metabolism and immunosuppression.

#### 3.2.3 Immune evasion and SE networks

Preventing HCC-specific immune evasion and overcoming resistance is vital for how TME influences HCC development and progression (Chen C. et al., 2023; Lu J. et al., 2024). The immune evasion process of HCC unveils the dynamic interaction of the immune microenvironment with abnormal metabolism and the dysregulated gut microbiome. Lu et al. previously revealed that silencing PAARH or up-regulating VEGF ameliorated the malignancy of the liver cancer cells and immune evasion. Functionally, PAARH increased the immune evasion capability of liver cancer cells by elevating VEGF expression to promote M2 macrophage polarization (Lu X. et al., 2024). Additionally, programmed death receptor-1/programmed cell death one ligand 1 (PD-1/PD-L1) checkpoint inhibitors are promising treatments in advanced HCC nowadays and in the future, which is also involved in immune evasion (Li et al., 2022; Hao et al., 2023). Apart from the experimental result, in the prognostic model based on SE-related genes, higher infiltration levels of M0 macrophages and upregulated CTLA4 and PD1 in the high-risk group, implying that immunotherapy could be effective for those patients (Wei et al., 2023).

Importantly, the networks between SEs and immune components are pivotal during liver cancer development. Aberrant SE networks drive the overexpression of immunosuppressive molecules. According to Cao’s research, expression correlation analysis was performed by the Tumor Immune Estimation Resource web server. TEAD2, TEAD3, NRF1, HINFP and TCFL5 were identified as potential transcription factors for HCC-specific SE-controlled genes associated to oxidative stress response (Liu B. et al., 2024). The five transcription factors were positively correlated with SPIDR expression, with the highest correlation coefficient for NRF1. NRF1 and SPIDR expression was upregulated in HCC tissues and cells. NRF1 elevated SPIDR transcription by combing to its SE.

Additionally, SEs regulate the secretion of cytokines and chemokines that modulate immune cell infiltration. In HCC models, SE-mediated activation of TGF-β signaling promotes the recruitment of immunosuppressive cells, including Tregs and tumor-associated macrophages (TAMs), while suppressing cytotoxic CD8^+^ T-cell activity (Du et al., 2022). This immunosuppressive milieu is further reinforced by SE-driven expression of VEGF-A, which enhances angiogenesis and establishes a hypoxic TME conducive to tumor progression (Pope et al., 2019). Furthermore, SEs contribute to ECM remodeling by upregulating matrix metalloproteinases (MMPs) and fibronectin, facilitating cancer cell invasion and metastasis. For example, the SE-associated gene MMP9 is overexpressed in HCC tissues and correlates with poor prognosis, likely due to its role in degrading basement membranes and promoting vascular invasion (Hu et al., 2024).

However, deeper mechanisms between SE network and HCC immune alterations need further basic researches.

### 3.3 Super-enhancer driven hallmarks of cancer in HCC

SEs contribute to the hallmarks of cancer, the biological capabilities that are acquired during tumorigenesis to sustain growth, resistance to death, and invasiveness. In HCC, SEs are linked to the following hallmark capabilities.

#### 3.3.1 Uncontrolled proliferation

SEs drive gene expression in the cell cycle and proliferation in several somatic tumors, such as melanoma and head and neck squamous cell carcinoma (Cai et al., 2024; Berico et al., 2023; Maezawa et al., 2020). Similarly, in liver cancer, SEs promote continuous cell division and tumor growth by enhancing the expression of key cell cycle regulators, such as cyclins and cyclin-dependent kinases (CDKs). He’s group utilized epigenomic profiling of DHX37-knockdown and control HCC cells, indicating that DHX37 is associated with SE’s activity. At the same time, DHX37 was deemed one of the top RNA helicase-related upregulated genes in HCC. Under this phenomenon, co-occupation of its promoter and SE elements takes a pivotal effect on the interaction DHX37 with pleiotropic regulator 1 (PLRG1), increasing cyclin D1 (CCND1) expression in transcriptional levels (Liu Z. et al., 2022).

#### 3.3.2 EMT and metastasis

Up to now, SEs have been verified to be associated with the EMT and metastatic nature of somatic tumors, especially in head and neck squamous cell carcinoma (Zhang et al., 2021), gastric cancer (Jin et al., 2023), CRC (Yu et al., 2021), and even liver cancer (Su et al., 2023).

In Wang’s study, 17 endoplasmic reticulum stress (ERS)-related SEs were identified by comparing ERS-treated HCC cells with untreated controls through ChIP-seq and RNA-seq. Using CRISPR-Cas9 and RT-qPCR, CAMP-responsive element binding protein 5 (CREB5) was identified as a key target of ERS-related SE. ERS activation increased the expression of several EMT markers by modulating the expression of CREB5. Also, CREB5 promoted epithelial-mesenchymal transition (EMT) in liver cancer cells by directly binding to the promoter region of tenascin-C (TNC) and upregulating its transcription (Wang et al., 2025).

Significantly, SE-lncRNAs can interact with their associated enhancer regions in cis and modulate the expression of oncogenes or key signal pathways in liver cancer (Song et al., 2024). Previously, Su’s group identified and characterized a novel SE-associated lncRNA—LINC01004, a crucial oncogene in HCC. LINC01004 promoted cell proliferation and metastasis of HCC, the expression of which could be mediated by E2F1 to the SE (Li et al., 2023). More recently, Su et al. identified another SE-driven LncRNA—LINC01089, forming an epigenetic network to promote HCC metastasis. Mechanically, LINC01089 activated ERK signaling and EMT by modulating DIAPH3 alternative splicing, which can inhibit N6-methyladenosine-mediated mRNA stabilization. The loss of LINC01089 elevated the expression of the DIAPH3 protein level, which inhibited the ERK/Elk1/Snail axis and EMT of HCC cells (Su et al., 2023).

Interestingly, in HBV-related HCC, Zhang et al. first confirmed that ETV4 is significantly upregulated by Hepatitis B virus protein (HBx) and involved in SE-associated chromatin accessibility (Zheng et al., 2022). Upregulated expression of ETV4 promotes HCC cell invasion and metastasis by upregulating DVL2. This study provided insight into the SEs that could participate in the ETV4-DVL2-β-catenin axis, which is especially helpful for treating patients with aggressive HBV-related HCC.

Emerging evidence underscores the critical involvement of SEs in hepatic metastasis beyond their established role in metastatic liver cancer. CDK12 has been established as a key driver of direct hepatic metastasis in CRC, with pharmacological inhibition demonstrating potent suppression of CRC cell proliferation, survival, and stemness maintenance. Mechanistic interrogation reveals that CDK12 ablation preferentially disrupts transcription initiation at SE-associated loci. Two SE-associated oncogenes—BCL2L1 and CCDC137—were identified as central mediators of metastatic progression through integrated analysis of super-enhancer landscapes and RNA sequencing data. These genes orchestrate a pro-metastatic program by coordinately enhancing cellular survival, proliferative capacity, and stemness acquisition, thereby significantly increasing hepatic metastatic propensity in CRC models (Dai et al., 2022).

Notably, transcriptional regulation in liver metastases exhibits unique features distinct from primary CRC and HCC. Pioneering work through the patient-derived xenograft (PDX) model by Zhang et al. revealed hepatocyte nuclear factor 1-alpha (HNF1A) as a master regulator of metastasis-specific SEs. (Cai et al., 2021). Their analysis demonstrated a 3.6-fold enrichment of HNF1-binding motifs in liver metastasis-derived cell lines and a 2.8-fold upregulation of HNF1A in synchronous liver metastases vs localized tumors. This finding was corroborated by Teng et al., who further identified that liver-enriched transcription factors (LETFs), including FOXA2 and HNF1A, (1) bind to metastasis-associated enhancers (2) activate hepatic-specific gene networks (e.g., APOA2, CYP3A4), and (3) establish an ectopic liver-like microenvironment facilitating CRC colonization (Teng et al., 2020).

Additionally, the function of SEs in pancreatic cancer-originated metastasis liver cancer should be exploited. Herein, to comprehensively describe the landscapes in this phenomenon, Shen’s group has established a super-enhancer-related metastatic (SEMet) classifier based on 38 prognostic hepatic metastatic (HM) genes (Chen D. et al., 2023). Their SEMet classifier better predicted HM patients’ prognosis, distinct from other clinical traits and 33 published signatures. These signatures demonstrated that E2F7 may promote pancreatic cancer hepatic metastasis by upregulating TGM2 and DKK1. This novel design provided new insights into personalized treatment approaches in the clinical management of metastatic pancreatic cancer patients.

#### 3.3.3 Resistance to apoptosis

HCC progression is closely associated with dysregulated apoptotic pathways. Recent studies have revealed that SE-driven transcriptional networks are pivotal in orchestrating apoptosis resistance through dual regulatory mechanisms (Fabregat, 2009). As mentioned above, the upregulation of CREB5 substantially increased cell proliferation, migration, and invasion and promoted EMT, inhibiting HCC cell apoptosis (Wang et al., 2025). In the SE-related prognostic model, the CBX2 gene (a suppression gene) was included, and the expression was found to inhibit HCC cell viability and migration while promoting apoptosis (Wu et al., 2024). However, this field in HCC has not been comprehensively explored. Thus, the roles of SEs in the resistance to apoptosis need further experimental research.

### 3.4 SE-related risk model establishment

Nowadays, instead of probing into the SE itself, super-enhancer-related genes (SERGs) are equally critical in exploring cancer mechanisms. SERGs were utilized to explore the underlying landscapes induced by SEs in acute myeloid leukemia (Sang et al., 2022), glioma (Hu et al., 2023), pancreatic cancer (Chen D. et al., 2023), and breast cancer (Wu Q. et al., 2023). Similarly, Wei et al. downloaded SERGs from a super-enhancer database (SEdb) (Jiang et al., 2019). They obtained transcriptome analysis and related clinical information from the Tumor Cancer Genome Atlas (TCGA) and the International Cancer Genome Consortium (ICGC) databases. Then, multivariate Cox regression analysis was applied to construct a four-gene prognostic signature. The high-risk group displayed a worse prognosis, more M0 macrophage infiltration, and higher expression of CTLA4 and PD1 (Wei et al., 2023).

Xie’s group recently downloaded SERGs from SEdb (Jiang et al., 2019) to establish a stable HCC prognostic model. Subsequently, five genes—CBX2, TPX2, EFNA3, DNASE1L3, and SOCS2 were selected, while the high-risk group indicated a worse prognosis. Notably, tumor-associated pathological pathways were more enriched in the high-risk group. Among these genes, this study also validated that CBX2 downregulation inhibited HCC cell viability, migration, and cell cycle progression and promoted apoptosis (Wu et al., 2024). Thus, these SERG models theoretically provided more personalized therapeutic methods, especially the selection of immune chemotherapy inhibitors (ICIs) and targeted drug identification.

### 3.5 Translational and therapeutic roles of SEs in liver cancer

The oncogenic roles of SEs in liver cancer, position them as compelling therapeutic targets. Mechanistically, SE-driven activation of oncogenes (e.g., *MYC*, *AJUBA*) and dysregulated transcriptional complexes (e.g., BRD4/CDK7-MED1) provide actionable vulnerabilities. Preclinically, inhibitors targeting SE-associated components—such as BRD4 antagonists (e.g., AZD5153) and CDK7 inhibitors—effectively suppress SE-mediated oncogene transcription and tumor growth. CRISPR-based disruption of SEs or their regulatory TFs (e.g., *SETD1A*) further validates their therapeutic potential. Challenges like tumor heterogeneity and SE plasticity necessitate combination strategies, including synergizing SE-targeted therapies with immune checkpoint inhibitors or epigenetic modulators. Translational efforts now focus on biomarker-driven clinical trials to exploit SE-driven oncogene dependencies while minimizing off-target effects.

## 4 Mechanisms of super-enhancer dysregulation in liver cancer

SEs regulate the expression of critical genes in normal and cancerous cells. Still, their activity can become dysregulated in liver cancer due to epigenetic modifications, TF networks, and chromatin remodeling changes. These dysregulated SEs promote the expression of oncogenes and other cancer-associated genes, driving the malignant transformation and aggressive behavior of liver cancer cells.

### 4.1 Epigenetic modifications

Epigenetic reprogramming is a hallmark of SE dysregulation in HCC, involving aberrant histone modifications, DNA methylation changes, and chromatin accessibility remodeling.

#### 4.1.1 Histone modification imbalances

SE regions in HCC exhibit elevated levels due to the aberrant recruitment of histone acetyltransferases (HATs) such as p300/CBP (Liu Y. et al., 2024). This modification promotes chromatin relaxation and facilitates the assembly of transcriptional machinery at oncogenic SEs (e.g., MYC, CCND1). Loss of histone deacetylases (HDACs), which generally counteract acetylation, further amplifies SE-driven oncogene expression (Tu et al., 2024; Xue et al., 2023). SE-associated loci in HCC show increased H3K4me3 (an active promoter mark) and reduced H3K27me3 (a repressive Polycomb mark) (Chen J. et al., 2023). This imbalance creates a permissive chromatin state for TF binding, activating pro-metastatic genes like SNAI1 and TWIST1.

Alcohol-related hepatitis (AH) has been established as a significant risk factor for hepatocellular carcinoma development (Llovet et al., 2021; Wang et al., 2020). Mechanistically, AH induces neutrophil infiltration in hepatic tissues *via* cytokine pathway activation, which drives chemokine overexpression. Liu et al.‘s multi-omics analysis revealed SE formation at multiple CXCL loci (Liu et al., 2021). RNA-seq and histone modification ChIP-seq of human liver explants show upregulation of multiple CXCL chemokines in AH. Functional validation demonstrated that dCas9-KRAB-mediated SE disruption or pharmacological BET protein inhibition substantially reduced chemokine production *in vitro*. Importantly, murine AH models showed attenuated neutrophil infiltration following BET inhibition. These findings elucidate the pivotal role of SEs in amplifying inflammatory cascades through chemokine regulation, suggesting BET protein blockade as a promising therapeutic strategy for AH management.

#### 4.1.2 DNA hypomethylation at SE-proximal regions

The obtained DNA methylomes suggest that transcription factors regulate the local activity of SEs, while trans-acting factors modulate DNA methylation profiles, thereby influencing transformation processes during carcinogenesis (Heyn et al., 2016). Interestingly, in wild-type mice maintained on an *ad libitum* diet without supplementation, age-related hypomethylation predominantly localized to SEs of highly expressed liver-function genes (Cole et al., 2017). Genes with hypomethylated enhancers showed significant overlap with age-dependent transcriptional alterations. Hypermethylation concentrated within CpG islands bearing bivalent histone modifications (H3K4me3/H3K27me3), mirroring patterns observed in hepatic malignancies. These epigenetic aging signatures were significantly attenuated in Ames dwarf and calorie-restricted models, while rapamycin intervention exhibited more selective but less pronounced effects.

### 4.2 Transcriptional network hijacking

TFs are essential in regulating SEs’ activity, especially in somatic cancers (Whyte et al., 2013; Wang et al., 2023). In liver cancer, specific TFs are frequently overexpressed or mutated, leading to the aberrant activation of SEs and the related oncogenes they regulate (Craig et al., 2023; Yang S. et al., 2024; Benichou et al., 2024). These TFs work with other chromatin-associated proteins to reprogram the genome and enable the transcriptional reprogramming seen in HCC.

HSFs—heat shock transcription factors, as a typical TF family, play crucial roles in the development of malignancies (Puustinen and Sistonen, 2020; Huang et al., 2022). HSF1 expression is elevated in HCC and is linked to poor prognosis in several datasets (Liu Y. et al., 2024). HSF1 deduced liver cancer cell proliferation both *in vitro* and *in vivo*, partly through modulation of H3K27ac levels, influencing enhancer distribution. Moreover, their findings demonstrated that HSF1 is bound to its promoter and SE elements to transcriptionally activate MYCN expression, thereby promoting liver cancer cell proliferation.

In addition, HSAL3 has been identified as an uncharacterized SE-driven oncogenic lncRNA. The transcription factors HSF1 and HCFC1 activate it at a fundamental level through its associated SE. The expression of HSAL3 is upregulated in HCC samples. Moreover, a higher level of HSAL3 expression is associated with poorer overall survival in HCC patients, possibly through the mediation of the NOTCH signaling pathway. Notably, nanoparticles loaded with siHSAL3 exert anti-tumor effects on HCC both *in vitro* and *in vivo*. HSAL3 represents a novel SE-driven oncogenic lncRNA, and siHSAL3-loaded nanoparticles emerge as promising therapeutic candidates for HCC treatment. This study highlights the potential of targeting SE-driven oncogenic lncRNAs as a viable strategy for HCC therapy, providing valuable insights into developing more effective treatment approaches (Yuan et al., 2023).

Moreover, Sun’s group demonstrated that transcription factors Twist1 and YY1 form a functional complex with the histone acetyltransferase p300, establishing phase-separated transcriptional condensates at the SEs miR-9. These biomolecular hubs create a high-concentration microenvironment that drives miR-9 overexpression, ultimately enhancing the migratory and invasive capabilities of HCC cells and promoting malignant progression. Notably, the diabetes therapeutic metformin (Met) effectively disrupts these Twist1-YY1-p300 condensates, leading to significant downregulation of miR-9 expression and subsequent suppression of HCC malignancy (Meng et al., 2023).

Interestingly, ERS exerts its effects by suppressing the liver-identity (LIVER-ID) TF network, primarily through the rapid degradation of LIVER-ID TF proteins (Ajoolabady et al., 2023). This suppression is further amplified by the induction of the transcriptional repressor NFIL3, which cooperatively inhibits LIVER-ID gene expression. Depleting hepatic TFs disrupts regulatory regions characterized by dense co-occupancy of LIVER-ID TFs, leading to functional decommissioning of BRD4-associated super-enhancers that maintain hepatic identity. While transient repression of liver-specific programs is a physiological component of injury repair, the persistent imbalance between ERS signaling and LIVER-ID transcriptional activity correlates with pathological outcomes. Experimental evidence from murine acute liver injury models and human septic patient livers demonstrates that sustained ERS-LIVER-ID disequilibrium drives hepatic dysfunction (Dubois et al., 2020). However, this phenomenon needs verification in the occurrence and development of liver cancer.

### 4.3 Chromatin remodeling and super-enhancer regulation

Chromatin remodeling is another mechanism that influences super-enhancer activity in malignancies, especially in CRC and squamous cell carcinoma (Tasdemir et al., 2016; Wang et al., 2021; Yi et al., 2020). In normal cells, chromatin is organized to restrict gene expression to appropriate levels. In cancer cells, however, chromatin remodeling complexes are often altered to facilitate SE activation and support the transcription of oncogenes.

A study led by Fan et al. demonstrates that HBx significantly upregulates ETV4 expression, a transcription factor mechanistically linked to SE-mediated chromatin remodeling. Elevated ETV4 levels drive HCC progression by activating the DVL2/β-catenin signaling axis, thereby enhancing tumor cell invasion and metastasis (Zheng et al., 2022). Therefore, this novel perspective needs further investigation.

## 5 Current challenges and future perspectives

**FIGURE 3:**
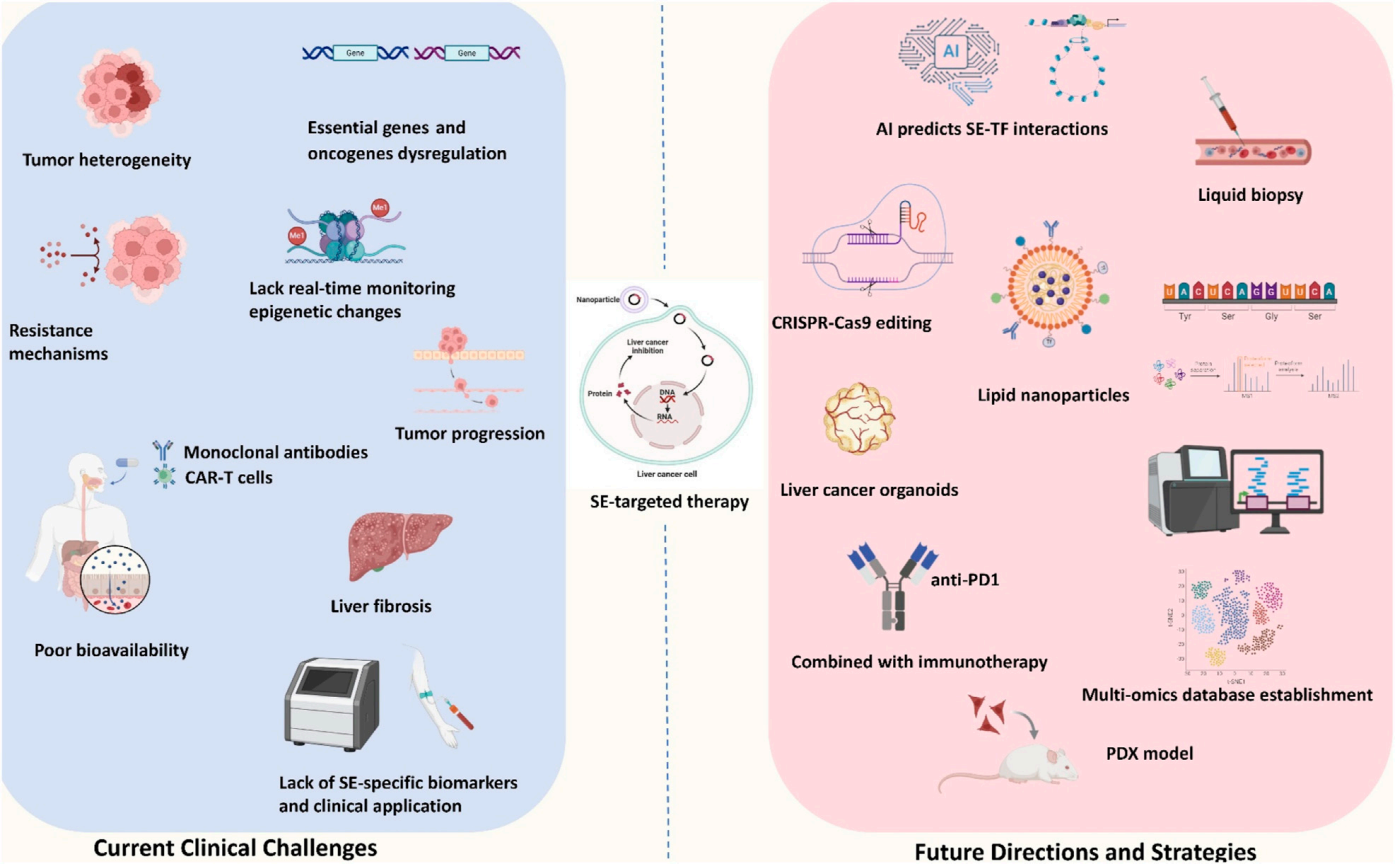
Current challenges and future perspectives of SE-target therapy. The current clinical challenges mainly included: tumor heterogeneity, unexplored drug resistance, lack of the real-time monitoring, poor bioavailability, potential liver fibrosis outcome and lack of SE-specific biomarkers and clinical applications. The future directions and strategies mainly included: AI predicts SE-TF interaction, CRISPR-Cas 9 editing, lipid nanoparticles, liver cancer organoid usage, PDX model, combination with immunotherapy and multi-omics database establishment.

Targeting SEs in liver cancer holds significant promise for therapeutic intervention, yet several challenges must be addressed to translate these therapies into clinical practice. Key challenges include ensuring specificity, minimizing toxicity, avoiding off-target effects, developing reliable biomarkers, and addressing the complexity of tumor heterogeneity. Additionally, the rapidly evolving field of epigenetic therapy and the potential for combination treatments necessitate a thorough exploration of prospects and the barriers that must be overcome. These challenges and future perspectives are summarized in Figure 3; Table 2.

**TABLE 2:** Current clinical challenges and future directions in SE-related therapy.

Category	Key points	Supporting evidence/Examples
Clinical challenges		
1. Scientific Challenges	**Tumor heterogeneity**: SEs vary across different periods and pathogenic types in liver cancer, complicating universal targeting strategies (Xue et al., 2022)	The dynamic regulating role of SE-driven oncogenic lncRNA-HSAL3 in HCC, which can be a potential target for inhibiting HCC progression (Yuan et al., 2023)
	**Off-target effects**: SEs regulate genes critical for normal liver function (e.g., metabolic pathways)	Inhibition of BRD4 disrupts both oncogenic and essential genes (Cazzanelli et al., 2024); HDACs promote the progression and reversal of liver fibrosis (Li et al., 2016)
2. Translational Hurdles	**Drug delivery limitations**: Poor bioavailability of SE-targeting agents	Hard to combine small molecule inhibitors with biological agents (mechanistic divergence, pharmacological incompatibility, and toxicity overlap), such as monoclonal antibodies or CAR-T cells (Peng et al., 2024)
	**Resistance mechanisms**: Cancer cells develop compensatory alteration post-treatment	Tumor cells may exploit feedback loops or compensatory mechanisms to overcome the effects of SE targeting (Mack et al., 2018). Lack of real-time monitoring of epigenetic changes during therapy period
3. Clinical Barriers	**Biomarker identification**: Lack of SE-specific biomarkers for patient stratification	H3K27ac and KLF5 levels correlate with SE activity but lack specificity for clinical use (Liu et al., 2024b; Li et al., 2024b)
Future Directions		
1. Technological Advances	High-resolution tools to dissect SE networks in patient cohorts	CRISPR-based SE modulation with immune checkpoint inhibitors (ICIs) (Jia et al., 2024)
	**AI-driven drug design**: Predict SE-TF interactions for precision targeting	Diffusion models (e.g., DeepConformer) enable rapid prediction of TF conformational changes, on SE-associated proteins that induce oncogenic transcriptional programs (Hamamoto et al., 2023)
2. Translational Strategies	**Combination therapies**: Pair SE inhibitors with immunotherapy (e.g., anti-PD1)	SE editing enhances tumor immunogenicity by upregulating antigen presentation and downregulating immunosuppressive factors (e.g., PD-L1), priming HCC for PD-1/PD-L1 (Shi et al., 2023)
	**Tissue-specific delivery:** Liver-tropic nanoparticles or viral vectors for SE modulator delivery	Lipid nanoparticles targeting hepatic SEs show preclinical promise (Yuan et al., 2023)
3. Clinical Collaboration	**Multi-omics databases’ establishment**: Integrate SE epigenomics with clinical outcomes in liver cancer trials	Combing transcriptomics, proteomics, genomics, epigenomics, single-cell, spital transcriptomics and FiTAc-seq) could lead to the identification comprehensive profile of SEs (Font-Tello et al., 2020)
	**Patient-derived models**: Organoids and PDX models to test SE-targeting therapies	PDX models unveils the tissue-specific transcription landscapes in liver metastasis of CRC, related the SE (Teng et al., 2020)

### 5.1 Specificity and off-target effects

A significant challenge in targeting super-enhancers is achieving specificity for cancer cells without affecting normal tissues. Super-enhancers regulate critical genes that are essential for cancer cell survival and normal cellular functions. This dual role complicates the development of therapies that selectively target cancer cells while sparing healthy ones, as unintended effects on normal cells could lead to significant toxicities.

Concerningly, proteins involved in SE regulation, such as BET proteins, p300, and HDACs, are also integral to normal cellular processes, including cell cycle regulation, differentiation, and immune responses (Cazzanelli et al., 2024; Li et al., 2016; Claveria-Cabello et al., 2020; Peng J. et al., 2019; Zhu S. et al., 2024). Inhibiting these proteins could result in off-target effects, potentially harming normal liver cells or other tissues.

Developing highly selective inhibitors that specifically target tumor-specific SEs or associated TFs is crucial to mitigate these risks. Advanced techniques such as small molecule inhibitors with enhanced selectivity, CRISPR/Cas9-based gene editing (Wang et al., 2022), and RNA interference (Chen et al., 2018) could target cancer-specific epigenetic machinery without disrupting normal cellular functions. Recent advances in deep learning models provide unprecedented tools to systematically map HCC-specific SE landscapes by integrating multi-omics data, including chromatin accessibility profiles, TF binding patterns, and 3D chromatin interactions. For instance, convolutional neural networks (CNNs) trained on chromatin accessibility data (e.g., ATAC-seq) and histone modification marks (H3K27ac, H3K4me1) can predict SE-driven oncogenic hubs with cell-type specificity.

### 5.2 Tumor heterogeneity and super-enhancer plasticity

Liver cancer, like many other cancers, exhibits significant tumor heterogeneity (Xue et al., 2022), both within and between tumors. Different tumor regions may display distinct genetic and epigenetic profiles, including variations in SE activation. This heterogeneity complicates the development of universal therapeutic approaches targeting SEs. Furthermore, the epigenetic plasticity of tumor cells—their ability to rapidly alter their epigenetic states in response to therapeutic pressure—can lead to the emergence of therapy-resistant subclones. Although SE-targeted therapeutic resistance has not been detected in liver cancer, this phenomenon has been verified in acute myeloid leukemia (Ottema et al., 2021). The dynamic nature of SE activity, combined with tumor heterogeneity, means that therapies targeting specific SEs may not be effective across all tumor subtypes or stages of disease progression. Tumor subclones may reprogram their super-enhancers in response to treatment, reducing the efficacy of therapies over time.

Personalized therapeutic strategies will be essential to address tumor heterogeneity. Single-cell transcriptomics and epigenomic profiling can identify specific SE signatures and epigenetic alterations in individual tumors, enabling more targeted therapies, especially in ovarian cancer (Lawrenson et al., 2019) and peripheral neuroblastic tumors (Yuan et al., 2022). Combination therapies that simultaneously target SEs and other oncogenic pathways (e.g., PI3K-AKT or Wnt/β-catenin) may help prevent resistance. Additionally, adaptive and specific therapeutic strategies that monitor changes in SE activity and adjust treatment regimens accordingly could improve outcomes.

### 5.3 Biomarker development and novel methods for patient stratification

A significant limitation in the current landscape of epigenetic therapies is the lack of reliable biomarkers to identify patients who are most likely to benefit from SE-targeted therapies. Since SE activity is context-dependent and varies across different cancer types, including liver cancer (Teng et al., 2020), predictive biomarkers are needed to determine which tumors exhibit aberrant super-enhancer regulation. They are likely to respond to such therapies. Identifying biomarkers associated with SE activity in liver cancer is complex. Unlike genetic mutations or protein expression markers, epigenetic modifications regulating super-enhancers are dynamic and vary widely among patients. Moreover, the expression of SE-associated genes may not always correlate with the degree of super-enhancer activation.

Advances in liquid biopsy technologies and next-generation sequencing (NGS) may enable real-time monitoring of SE-driven transcriptional networks, offering a non-invasive approach to assess SE activity and predict therapeutic responses. The development of epigenetic biomarkers, such as changes in TFs (e.g., KLF5) and histone modifications (e.g., H3K27ac or H3K4me1) (Liu Y. et al., 2024; Li et al., 2024b), could provide insights into tumor biology and guide the selection of targeted therapies for liver cancer (Bai et al., 2024). Additionally, integrating multi-omics data (e.g., genomics, epigenomics, proteomics, single-cell, and even spital transcriptomics) could lead to identifying composite biomarkers that offer a more comprehensive profile of SE activity and cancer progression. Additionally, FiTAc-seq generates high-quality enhancer landscapes and SE annotation in numerous archived FFPE samples from distinct tumor types (Font-Tello et al., 2020). This approach will be of great significance for both basic and clinical researchers, notably for liver cancer. Only by constructing more comprehensive SE-related transcriptomic, proteomic, and metabolic landscapes the diagnostic role of SEs in liver cancer can be deciphered deeply.

Patient-derived models, including organoids and PDX models, have been verified in somatic cancers (Antal et al., 2023; Li J. et al., 2024), notably in liver metastasis of CRC (Teng et al., 2020). Organoids retain tumor heterogeneity and microenvironment features, enabling high-throughput screening of SE inhibitors (e.g., BRD4 or CDK7) while preserving genomic and transcriptomic profiles. PDX models, transplanted into immunodeficient mice, provide *in vivo* validation of therapeutic efficacy and resistance mechanisms. These models also reveal SE-driven oncogene dependencies (e.g., MYC) and optimize dosing regimens for clinical translation. However, challenges remain in recapitulating immune interactions and addressing organoid scalability limitations.

Herein, we propose a systematic novel liquid biopsy panel [like in CRC (Nakamura et al., 2022)] targeting SE-associated epigenetic signatures for HCC detection and monitoring, integrating three key innovations: SE-driven biomarker selection, multi-modal detection technology, and clinical translation framework. This SE-centric liquid biopsy strategy addresses limitations in HCC diagnosis by capturing dynamic epigenetic alterations rather than static genetic mutations. Its modular design allows integration with existing biomarkers (e.g., AFP), potentially enabling early detection of premalignant SE activation during hepatocarcinogenesis.

### 5.4 Resistance mechanisms to super-enhancer targeting

Despite the promise of epigenetic therapies, resistance remains a significant challenge. Tumors may adapt to the loss of SE-driven gene expression by activating alternative transcriptional pathways or reprogramming chromatin structures. Resistance mechanisms could involve the activation of compensatory SEs that drive the expression of other oncogenes or the upregulation of alternative TFs that bypass the initial epigenetic blockade. Tumor cells may exploit feedback loops or compensatory mechanisms to overcome the effects of SE targeting (Chen et al., 2025; Mack et al., 2018). For example, inhibiting one SE could activate parallel oncogenic pathways or alternative super-enhancers driving the same cancer-associated genes.

Understanding resistance mechanisms will be critical for developing more durable therapies. A combinatorial approach that targets both SE machinery and other vital pathways, such as cell cycle regulation or immune checkpoints, may help prevent resistance. Real-time monitoring of epigenetic changes during therapy could allow clinicians to dynamically adjust treatment plans and to avoid resistance before it becomes clinically significant.

### 5.5 Clinical translation and safety concerns

While preclinical studies have demonstrated the potential of targeting SEs in liver cancer, translating these findings into clinical practice presents several challenges. Safety and toxicity remain key concerns, mainly due to the involvement of SE regulators in normal physiological processes. The broad roles of SE-associated proteins in maintaining normal cellular homeostasis raise concerns about off-target toxicity, especially in organs like the liver (Whyte et al., 2013). Additionally, the chronic nature of liver cancer therapy necessitates a thorough assessment of long-term safety in clinical trials. Herein, we summarize the clinical trials currently underway or have concluded in Table 3. This table summarizes key SE-driven oncogenic mechanisms in liver cancer, including SE-associated genes (e.g., AJUBA, MYC, SETD1A, HCCL5), their roles in proliferation, EMT, metabolic reprogramming, and immune evasion. It highlights therapeutic targets (e.g., BRD4, CDK7) and clinical implications, such as small-molecule inhibitors and gene-editing strategies for SE disruption. Recent advances in SE-targeted therapies for liver cancer have transitioned from preclinical validation to early-phase clinical trials. For instance, inhibitors targeting BRD4 (e.g., AZD5153) and CDK7 (e.g., SY-5609) are currently in Phase I/II trials (NCT04840589, NCT04247126), demonstrating preliminary efficacy in suppressing SE-driven oncogenes (MYC, SIRT7) and reducing tumor burden in advanced HCC. Additionally, BET inhibitors like OTX-015 have shown synergistic effects with immune checkpoint inhibitors (anti-PD-1) in preclinical models by disrupting immunosuppressive SE networks, supporting ongoing combinatorial trials (NCT05487170). Emerging CRISPR-based epigenome-editing tools, such as dCas9-p300, are being explored to selectively silence oncogenic SEs (e.g., HSF1-associated enhancers) in preclinical studies, offering precision modulation of transcriptional addiction. These trials highlight the dual strategy of pharmacologically disrupting SE complexes or editing SE architecture to overcome therapeutic resistance. Future efforts should prioritize biomarker-driven patient stratification to optimize SE-targeted regimens and address heterogeneity in HCC.

**TABLE 3:** SE-related potential drugs in clinical trials for liver cancer.

Drug name	Target	Mechanism profile	Clinical trial phase	Clinical translation	Therapeutic implications	NCT number
OTX015	BRD4	BET inhibitor disrupting SE-mediated oncogene transcription (e.g., MYC)	Phase I/II	Demonstrated tumor regression in HCC models; synergizes with chemotherapy (e.g., sorafenib)	Targets SE-driven transcriptional addiction; potential for MYC-driven HCC subtypes	NCT02259114
THZ1	CDK7	Covalent CDK7 inhibitor blocking SE-driven transcriptional addiction	Phase I	Suppressed SE-associated oncogenes (e.g., MYC) in preclinical models; limited toxicity in early trials	Addresses transcriptional “bursts” driven by SEs; combats resistance to BET inhibitors	NCT03134638
BAY 1143572	CDK9	Selective CDK9 inhibitor targeting SE-driven transcriptional elongation	Phase I	Anti-proliferative effects in HCC cell lines; ongoing trials in solid tumors	Targets transcriptional elongation dependency; may overcome adaptive SE rewiring	NCT02345382
Dinaciclib	CDK9/CDK12	Pan-CDK inhibitor suppressing SE-associated transcriptional dependencies	Phase II	Reduced tumor burden in advanced HCC; efficacy correlates with MYC expression levels	Broad CDK targeting may mitigate compensatory SE activation; higher toxicity risk	NCT01676753

By integrating multi-omics data (genomic, epigenomic, and proteomic) with deep learning algorithms, artificial intelligence (AI) models can identify critical SE-TF regulatory nodes and dynamic binding patterns (Hamamoto et al., 2023). For instance, advanced frameworks like diffusion models (e.g., DeepConformer) enable rapid prediction of TF conformational changes, revealing cryptic binding pockets on SE-associated proteins that drive oncogenic transcriptional programs (Du et al., 2024). This approach accelerates target discovery 10-fold compared to traditional methods, while machine learning-powered virtual screening optimizes compound libraries to block pathological SE-TF networks. However, challenges remain in validating AI-predicted interactions through wet-lab experiments and addressing tumor heterogeneity in clinical translation.

Recent advances in liver cancer therapy have highlighted the potential of targeting SEs—critical regulatory elements driving oncogene expression—to reshape the tumor microenvironment and amplify immunotherapy efficacy. Our proposed “SE Editing + Immunotherapy” combination strategy integrates CRISPR-based SE modulation [similar to multiple myeloma (Li J. et al., 2024)] with immune checkpoint inhibitors (ICIs) in breast cancer, colorectal cancer, and other cancers (Jia et al., 2024), demonstrating remarkable clinical benefits in advanced HCC. By selectively disrupting oncogenic SEs (e.g., those activating HSAL3 or miR-9), this approach suppresses tumor-promoting pathways (e.g., NOTCH, β-catenin) while reducing immune evasion signals (Yuan et al., 2023). SE editing enhances tumor immunogenicity by upregulating antigen presentation and downregulating immunosuppressive factors (e.g., PD-L1), priming HCC for PD-1/PD-L1 inhibitors like nivolumab or pembrolizumab (Shi et al., 2023). Ongoing trials are exploring SE modulation with dual ICIs (e.g., anti-PD-1 + anti-CTLA-4) to further boost durable responses.

The development of targeted delivery systems, such as nanoparticles or antibody-drug conjugates (ADCs), could minimize systemic toxicity and improve drug localization to liver cancer cells. Combining small molecule inhibitors with biological agents, such as monoclonal antibodies or CAR-T cells (Chakraborty and Sarkar, 2022; Peng et al., 2024), may offer synergistic benefits while reducing adverse effects. Thus, these therapeutic strategies aim to disrupt the specific SE-driven transcriptional programs that sustain cancer cell survival and progression, offering the potential for personalized treatment options that are more effective and less toxic than traditional chemotherapy.

To address these issues, in 2019, 102,373 *cis*-regulatory elements were identified in the pig liver, mainly including enhancers and SEs, highlighting 26 core transcription regulatory factors in the pig liver as well (Luan et al., 2019). Intriguingly, Pan’s group has established the SE repertoire in porcine liver (Zhang et al., 2023). In this database, the expression profiles were mainly sourced from fetal and 70-day-old pigs, demonstrating that many SE-associated genes were positively related to hepatic metabolisms and detoxification pathways. Their results illustrated the disparity and linkage between promoter and enhancer markers. Considering the similarity between porcine and human beings, more SE-related mechanisms in liver cancer can be identified, providing more robust evidence for targeted therapy, especially for the unknown side effects.

### 5.6 Innovative perspectives and emerging frontiers in super enhancer-driven liver cancer research

While the current review synthesizes established mechanisms linking SEs to HCC progression, advancing the field requires a paradigm shift toward innovative frameworks and unexplored intersections. Recent breakthroughs in single-cell epigenomics (Gu et al., 2025; Lewis et al., 2024; Liu S. et al., 2024), spatial transcriptomics (Mahat et al., 2024; Manicardi et al., 2023), and CRISPR-based functional screens (Xi et al., 2025; Kasprzyk et al., 2024) have unveiled unprecedented heterogeneity in SE landscapes across variable malignancies. For instance, emerging studies highlight dynamic SE rewiring during therapy resistance, where subpopulations of tumor cells exploit SE plasticity to activate compensatory oncogenic pathways. Integrating these findings, we propose that SE-driven transcriptional addiction in HCC could be leveraged to design “epigenetic vulnerability” screens. By mapping SE-associated dependencies in treatment-naïve *versus* resistant tumors, researchers may identify context-specific targets, such as SE-regulated non-coding RNAs or metabolic gatekeepers, that circumvent traditional oncogene-centric limitations. Furthermore, leveraging AI to predict SE connectivity networks—incorporating multi-omics data from circulating tumor DNA, liquid biopsies, and patient-derived organoids—could enable real-time monitoring of SE activity as a biomarker for therapeutic response (Kim et al., 2025). In the future, our own study will focus on the SE-related multi-omics in HCC, integrating these innovative technologies.

A second frontier lies in decoding the interplay between SEs and the TME. SEs in stromal or immune cells (e.g., tumor-associated macrophages) may indirectly promote malignant tumor progression by regulating cytokine secretion or immune checkpoint expression (Zhang T. et al., 2022; Betancur et al., 2017). Additionally, the gut-liver axis represents an untapped dimension: microbiome-derived metabolites like butyrate modulate histone acetylation patterns, potentially reshaping SE architectures in pre-malignant hepatocytes. Future studies should explore whether SE inhibition synergizes with microbiota modulation or immune checkpoint blockade to achieve durable remission. By embracing these multidimensional approaches—spanning SE-TME interactions, AI-driven biomarker discovery, and metabolite-epigenome crosstalk—the field can transition from descriptive SE mapping to mechanistically innovative, clinically transformative strategies.

## 6 Conclusion

The therapeutic potential of targeting SEs in liver cancer represents a promising frontier in oncology. By disrupting the epigenetic and transcriptional networks that drive liver cancer progression, we could reprogram the cancer cell transcriptome, thereby inhibiting tumor growth and metastasis. However, significant challenges remain, including specificity, toxicity, tumor heterogeneity, and the need for reliable biomarkers. Combination therapies targeting super-enhancer activity and other oncogenic signaling pathways could offer the most effective strategy for overcoming these challenges.

As we continue to deepen our understanding of SE biology and its role in liver cancer, developing more targeted and personalized therapies will be crucial in translating these findings into effective clinical treatments. Future research must address the complexity of SE regulation, identify biomarkers for patient stratification, and refine combination treatment approaches to ensure that liver cancer patients benefit from the full potential of epigenetic therapies.

## Glossary

ACACA: acetyl-CoA carboxylase 1
ADC: antibody-drug conjugates
AH: Alcohol-related hepatitis
AJUBA: ajuba LIM protein
ALD: alcohol-associated liver disease
BRD4: bromodomain-containing protein 4
CCND1: cyclin D1
CCT: phosphocholine cytidylyltransferase
CDKs: cyclin-dependent kinases
CHC: combined hepatocellular-cholangiocarcinoma
CHKA: choline kinase-α
CNNs: convolutional neural networks
CRC: colorectal cancer
CREB5: CAMP responsive element binding protein 5
EMT: epithelial–mesenchymal transition
ERS: endoplasmic reticulum stress
FA: fatty acid
FASRL: synthesis-related lncRNA
FLHS: fatty liver haemorrhage syndrome
GO: Gene Ontology
H3K4me1: histone H3 lysine 4 mono methylation
H3K27ac: acetylation of histone H3 at lysine 27
HATs: histone acetyltransferases
HBV: hepatitis B virus
HBx: Hepatitis B virus protein
HCC: hepatocellular carcinoma
HCV: chronic hepatitis C virus
HDACs: histone deacetylases
HFD: high-fat diet
HLP: hyperlipidemia
HM: hepatic metastatic
HNF1A: hepatocyte nuclear factor 1-alpha
HSF1: heat shock transcription factor 1
ICC: intra-hepatic cholangiocarcinoma
ICGC: International Cancer Genome Consortium
ICIs: immune chemotherapy inhibitors
KEGG: Kyoto Encyclopedia of Genes and Genomes
LETFs: liver-enriched transcription factors
LIVER-ID: liver-identity
LLPS: liquid–liquid phase separation
LncRNAs: Long non-coding RNAs
MASLD: metabolic dysfunction-associated steatotic liver disease
MED: mediator complex
PDX: patient-derived xenograft
PLC: primary liver cancer
PLRG1: pleiotropic regulator 1
ROS: oxidative stress response
pri-miRNAs: primary microRNAs
SAM: S-adenosylmethionine
SEs: super-enhancers
SEdb: super-enhancer database
SEMet: super-enhancer-related metastatic
SERGs: super-enhancer-related genes
SETD1A: SET domain containing 1A
SIRT7: Sirtuin 7
SOD: superoxide dismutase
TACE: trans-arterial chemoembolization
TAD: topologically associating domain
TARE: trans-arterial radioembolization
TCF4: transcription factor 4
TCGA: Tumor Cancer Genome Atlas
TEs: typical enhancers
TFs: transcription factors
TNC: tenascin-C
USF1: upstream stimulatory factor 1
